# Effect of Stent Inflation Pressure and Post-Dilatation on the Outcome of Coronary Artery Intervention. A Report of More than 90 000 Stent Implantations

**DOI:** 10.1371/journal.pone.0056348

**Published:** 2013-02-13

**Authors:** Ole Fröbert, Giovanna Sarno, Stefan K. James, Nawsad Saleh, Bo Lagerqvist

**Affiliations:** 1 Department of Cardiology, Örebro University Hospital, Örebro, Sweden; 2 Institution of Medical Sciences, Uppsala University, Uppsala, Sweden; 3 Department of Cardiology, Karolinska Hospital, Stockholm, Sweden; University Medical Center Utrecht, The Netherlands

## Abstract

**Background:**

Percutaneous coronary intervention (PCI) stent inflation pressure correlates to angiographic lumen improvement and stent expansion but the relation to outcome is not clarified. Using comprehensive registry data our aim was to evaluate how stent inflation pressure influences restenosis, stent thrombosis and death following PCI.

**Methods:**

We evaluated all consecutive coronary stent implantations in Sweden during 46 months from 2008 using data from the Swedish Coronary Angiography and Angioplasty Registry (SCAAR). We used logistic regression and Cox proportional hazard modeling to estimate risk of outcomes with different balloon pressures.

**Results:**

In total, 93 697 stents were eligible for analysis and divided into five different pressure interval groups: ≤15 atm, 16–17 atm, 18–19 atm, 20–21 atm and ≥22 atm. The risks of stent thrombosis and restenosis were significantly higher in the ≤15 atm, 18–19 atm and ≥22 atm groups (but not in the 16–17 atm group) compared to the 20–21 atm group. There were no differences in mortality. Post-dilatation was associated with a higher restenosis risk ratio (RR) of 1.22 (95% confidence interval (CI) 1.14–1.32, P<0.001) but stent thrombosis did not differ statistically between procedures with or without post-dilatation. The risk of death was lower following post-dilatation (RR 0.81 (CI 0.71–0.93) P = 0.003) and the difference compared to no post-dilatation was seen immediately after PCI.

**Conclusion:**

Our retrospective study of stent inflation pressure identified a possible biological pattern—the risks of stent thrombosis and of restenosis appeared to be higher with low and very high pressures. Post-dilatation might increase restenosis risk.

## Background

Since the introduction of coronary balloon angioplasty (PCI) more than 30 years ago the concept has changed little: a fluid-filled balloon is advanced into a stenosed coronary artery segment and inflated with incompressible fluid thus dilating the artery and improving arterial patency and myocardial perfusion. Before the introduction of coronary stents, PCI was a trade-off between increasing luminal diameter at the site of a stenosis and common procedural complications such as mural thrombus, dissection and medial injury which all increased in frequency in animal models with balloon inflation pressure [1].

Stents changed this and using intravascular ultrasound (IVUS) it was soon discovered that optimization of stent expansion [2] and avoidance of stent thrombosis could be achieved with higher stent inflation pressures [3],[4]. However, such observations did not translate into a clinical benefit. In a study of 934 patients receiving bare metal stents, subjects were randomized to low (8–13 atmospheres (atm)) or high (15 to 20 atm) balloon pressure dilatation [5] but there was no difference between groups in survival or restenosis at 6-months angiographic follow-up. However, non-Q-wave myocardial infarction occurred almost twice as often in the high-pressure group. Using IVUS, a smaller randomized study demonstrated greater bare metal stent expansion after high-pressure dilatation initially and at 6-months follow-up but there was no difference in restenosis or target vessel revascularization rate between the high- or low pressure groups [6].

Malapposition and underexpansion of stents are associated with complications – first of all stent thrombosis. Post-dilatation with a non-compliant (NC) balloon as opposed to a stent-mounted semi-compliant balloon theoretically assures a more uniform distribution of wall stress and stent expansion and axial stent symmetry indices improve [7]. However, findings deviate and more optimal stent expansion with stent balloons than NC balloons has also been found [8]. The clinical benefit of high pressure post-dilatation remains unclarified and might even result in more intimal hyperplasia compared to a less aggressive approach [9].

Real world data are of paramount importance when different treatment strategies are evaluated. This is especially true for coronary stents, which are very often used “off-label” when the implantation takes place outside the scope of the approved indication. We evaluated death, stent occlusion and restenosis rate in relation to the applied stent pressure in all patients treated by coronary artery stent implantation during 46 months from 2008 and onwards, as recorded in the Swedish Coronary Angiography and Angioplasty Registry (SCAAR).

## Methods

### Study population

Our study included all patients in Sweden who had received coronary stents from January 1, 2008, to October 26, 2011. The analyses were based on maximal stent inflation pressure at the first recorded procedure during this time period.

### The SCAAR data

SCAAR, which is a part of the national SWEDEHEART registry, holds data on all consecutive patients from all centers (n = 29) that perform coronary angiography and PCI in Sweden. The registry is sponsored by the Swedish Health Authorities and is independent of commercial funding. The technology is developed and administered by the Uppsala Clinical Research Center. Since 2001, SCAAR has been Internet-based, with recording of data online through a Web interface in the catheterization laboratory; data are transferred in an encrypted format to a central server at the Uppsala Clinical Research Center. Information on restenosis in any previously implanted stent anywhere in Sweden has been registered for patients undergoing any subsequent coronary angiography on a clinical indication since March 1, 2004 and information on stent thrombosis since May 1, 2005. Long-term follow-up was obtained by merging the SCAAR database with other national registries based on all Swedish citizens' unique 10-digit personal identification number. Vital status and date of death was obtained from the National Population registry and was available until October 15, 2011. Merging of the registries was performed by the Epidemiologic Centre of the Swedish National board of Health and Welfare and approved by the ethics committee at Uppsala University. Because the data are anonymised written informed consent from each patient was not needed. Monitoring and verification of registry data have been performed in all hospitals since 2001 by comparing 50 entered variables in 20 randomly selected interventions per hospital and year with the patients' hospital records. The overall correspondence of data during the study period was 95.2%.

### Definitions

In the results, “stent inflation pressure” refers to the maximal inflation pressure in atm used when a stent was deployed. “Post-dilatation” refers to one or more additional dilatations within the stent area following stent deployment. An acute definite stent thrombosis is defined in SCAAR as an angiographic occlusion of a previously implanted stent with an acute clinical presentation [10]. A restenosis is defined as a clinically relevant angiographic stenosis in a previously implanted stent assessed by visual estimate (>50% diameter stenosis) or as a significant reduction in fractional flow reserve [11].

### Statistical analysis

Baseline characteristics were summarized with means and standard deviations for continuous variables and percentages for discrete variables. Cumulative event rates were estimated by the Kaplan-Meier method. The primary outcome variables were mortality, restenosis and stent thrombosis. To compensate for the non-randomized design of the study a Cox proportional hazard regression model was used to compare the risk of outcomes with different balloon pressures. All these variables were forced into the model: age, gender, diabetes, smoking, hypertension, hyperlipidemia, indication for angiography, angiographical finding, previous PCI, previous CABG, previous myocardial infarction, number of stents used, year of procedure, hospital, diameter and length of the stent, type of stent (drug-eluting or bare metal), stent brand, chronic total occlusion, classification of stenosis (A, B1, B2 or C), anatomical localization of lesion, restenotic lesion, bifurcation and use of post-dilatation. Mortality was calculated only in patients receiving a single stent. Calculations of the incidences of stent thrombosis and restenosis were performed with a focus on individual stents. The data are therefore presented from the stent perspective with patient and procedure data linked to the individual stents. To test for statistical interaction between balloon pressure and indication for PCI, between balloon pressure and type of stent (drug-eluting or bare metal) and between balloon pressure and post-dilatation/no post-dilatation, the following interaction terms were entered separately into the different statistical models: pressuregroup*indication, pressuregroup*type of stent and pressuregroup*post-dilatation. To compare categorical data of clinical and treatment relevance, we used the chi-square test. All reported P-values are two-sided. All analyses were done with SPSS statistical software, version 20.0 (Chicago, IL). A P-value of less than 0.05 was considered statistically significant.

## Results

### Patient and procedure characteristics

During the study period 94 342 stents were used, 645 were excluded due to incomplete data, leaving 93 697 stents eligible for analysis. We divided the material into five different groups representing a compromise between the number of stents per group and clinical relevance: ≤15 atm, 16–17 atm, 18–19 atm, 20–21 atm and ≥22 atm. In Tables 1 and 2 baseline and procedural variables are listed. Many variables were numerically almost identical. However, more men and higher proportions of risk factors such as diabetes mellitus, hypertension and hyperlipidemia were found in the high pressure groups (Table 1). Moreover, bivalirudin was very often used in association with stents in the ≤15 atm pressure group while heparins were more often used in the high pressure groups (Table 2). Also the use of drug-eluting stents and post-dilatation were more prevalent in the high pressure groups. Follow-up time was approximately 2 years for all groups (Table 2).

**Table 1:** Baseline characteristics.

*Baseline characteristics*	≤15 atm	16–17 atm	18–19 atm	20–21 atm	≥22 atm
Stents - no. (% of total)	14218 (15.2)	16022 (17.1)	21194 (22.6)	27129 (29.0)	15134 (16.2)
Age - yr. Mean (± SD)	67.3 (11.2)	67.1 (11.1)	66.9 (11.0)	67.1 (10.8)	67.3 (10.7)
Female sex - no. (%)	4188 (29.5)	4396 (27.4)	5576 (26.3)	6772 (25.0)	3735 (24.7)
Male sex - no. (%)	10030 (70.5)	11626 (72.6)	15618 (73.7)	20357 (75.0)	11399 (75.3)
Indication - no. (%)					
Stable coronary artery disease	2892 (20.3)	3585 (22.4)	5255 (24.8)	6971 (25.7)	4175 (27.6)
Unstable coronary artery disease	6748 (47.5)	7864 (49.1)	10287 (48.5)	13210 (48.7)	7173 (47.4)
STEMI	4206 (29.6)	4208 (26.3)	5099 (24.1)	6209 (22.9)	3360 (22.2)
Other	372 (2.6)	365 (2.3)	563 (2.7)	739 (2.7)	426 (2.8)
Diabetes mellitus - no. (%)					
Insulin treatment	1158 (8.1)	1350 (8.4)	1987 (9.4)	2609 (9.6)	1556 (10.3)
Non-insulin treatment	1396 (9.8)	1681 (10.5)	2359 (11.1)	3038 (11.2)	1761 (11.6)
Smoking status - no. (%)					
Never smoked	5570 (39.2)	6412 (40.0)	7909 (37.3)	10318 (38.0)	5646 (37.3)
Former smoker	4741 (33.3)	5545 (34.6)	7740 (36.5)	10187 (37.6)	5895 (39.0)
Current smoker	2622 (18.4)	3089 (19.3)	4274 (20.2)	5276 (19.4)	2933 (19.4)
Unknown	1285 (9.0)	976 (6.1)	1271 (6.0)	1348 (5.0)	660 (4.4)
Hyperlipidemia - no. (%)	6926 (48.7)	8014 (50.0)	11105 (52.4)	14882 (54.9)	8642 (57.1)
Hypertension - no. (%)	7736 (54.4)	9047 (56.5)	12325 (58.2)	16020 (59.1)	9176 (60.6)
Previous myocardial infarction - no. (%)	3530 (24.8)	4359 (27.2)	6034 (28.5)	7995 (29.5)	4977 (32.9)
Previous coronary artery by-pass grafting - no. (%)	1327 (9.3)	1511 (9.4)	2122 (10.0)	3005 (11.1)	1849 (12.2)

All information in the table is given “per stent”. Abbreviations: atm: atmosphere, STEMI: ST-segment elevation myocardial infarction.

**Table 2:** PCI data.

*PCI data*	≤15 atm	16–17 atm	18–19 atm	20–21 atm	≥22 atm
*Pre-procedure medication*					
Acetylsalicylic acid - no. (%)	13073 (91.9)	14938 (93.2)	19888 (93.8)	25628 (94.5)	14271 (94.3)
Clopidogrel - no. (%)	11569 (81.3)	13565 (84.7)	18136 (85.6)	23596 (87.0)	13111 (86.6)
Warfarin - no. (%)	311 (2.2)	331 (2.1)	462 (2.2)	572 (2.1)	302 (2.0)
*Procedure-related medication*					
Heparin - no. (%)	6819 (48.0)	9872 (61.6)	13640 (64.4)	18751 (69.1)	10454 (69.1)
LMWH - no. (%)	719 (5.1)	1474 (9.2)	2237 (10.6)	2064 (7.6)	1301 (8.6)
Bivalirudin - no. (%)	6547 (46.0)	4502 (28.1)	5091 (24.0)	6036 (22.2)	3188 (21.1)
Glucoprotein IIb/IIIa inhibitor - no. (%)	2399 (16.9)	2941 (18.4)	3345 (15.8)	3857 (14.2)	2134 (14.1)
*Stented artery - no (%)*					
De novo lesion	13698 (96.3)	15413 (96.2)	20201 (95.3)	25677 (94.6)	13920 (92.0)
Restenosis	107 (0.8)	129 (0.8)	181 (0.9)	233 (0.9)	180 (1.2)
In-stent restenosis	413 (2.9)	480 (3.0)	812 (3.8)	1219 (4.5)	1034 (6.8)
*ACC/AHA lesion class - no (%)*					
A	1656 (11.6)	1781 (11.1)	2273 (10.7)	2703 (10.4)	1202 (7.9)
B1	5251 (36.9)	6170 (38.5)	7580 (35.8)	9583 (35.3)	4712 (31.1)
B2	4819 (33.9)	5264 (32.9)	6875 (32.4)	8813 (32.5)	5366 (35.5)
C	2492 (17.5)	2807 (17.5)	4466 (21.1)	6030 (22.2)	3854 (25.5)
*Stented artery - no (%)*					
RCA	3767 (26.5)	4516 (28.2)	6514 (30.7)	9068 (33.4)	5392 (35.6)
LM	212 (1.5)	239 (1.5)	375 (1.8)	670 (2.5)	571 (3.8)
LAD	6071 (42.7)	6788 (42.4)	8855 (41.8)	11100 (40.9)	6163 (40.7)
LCx	3719 (26.2)	3981 (24.8)	4769 (22.5)	5412 (19.9)	2471 (16.3)
Radial artery graft	25 (0.2)	25 (0.2)	29 (0.1)	33 (0.1)	13 (0.1)
Saphenous vein graft	424 (3.0)	473 (3.0)	652 (3.1)	846 (3.1)	524 (3.5)
*Type of stent - no (%)*					
Bare metal stent implantation	9856 (69.3)	9864 (61.6)	12534 (59.1)	15451 (57.0)	7721 (51.0)
Drug-eluting stent implantation	4362 (30.7)	6158 (38.4)	8660 (40.9)	11678 (43.0)	7413 (49.0)
*Post-dilatation - no (%)*					
Post-dilatation	2238 (15.7)	3469 (21.7)	6327 (29.9)	10311 (38.0)	7612 (50.3)
No post-dilatation	11980 (84.3)	12553 (78.3)	14867 (70.1)	16818 (62.0)	7522 (49.7)
Stent diameter (mm). Mean (± SD)	3.00 (0.54)	3.04 (0.51)	3.05 (0.51)	3.07 (0.52)	3.12 (0.51)
Stent length (mm). Mean (± SD)	16.9 (5.9)	17.4 (6.0)	17.9 (6.3)	18.5 (6.8)	18.8 (7.0)
Chronic total occlusion - no (%)	372 (2.6)	374 (2.3)	597 (2.8)	887 (3.3)	616 (4.1)
Follow-up time (days). Mean (± SD)	734 (413)	724 (407)	699 (397)	657 (397)	681 (398)

All information in the table is given “per stent”. Abbreviations: atm: atmosphere, LMVH: Low molecular weight heparin.

### Stent thrombosis

**Figure 1:**
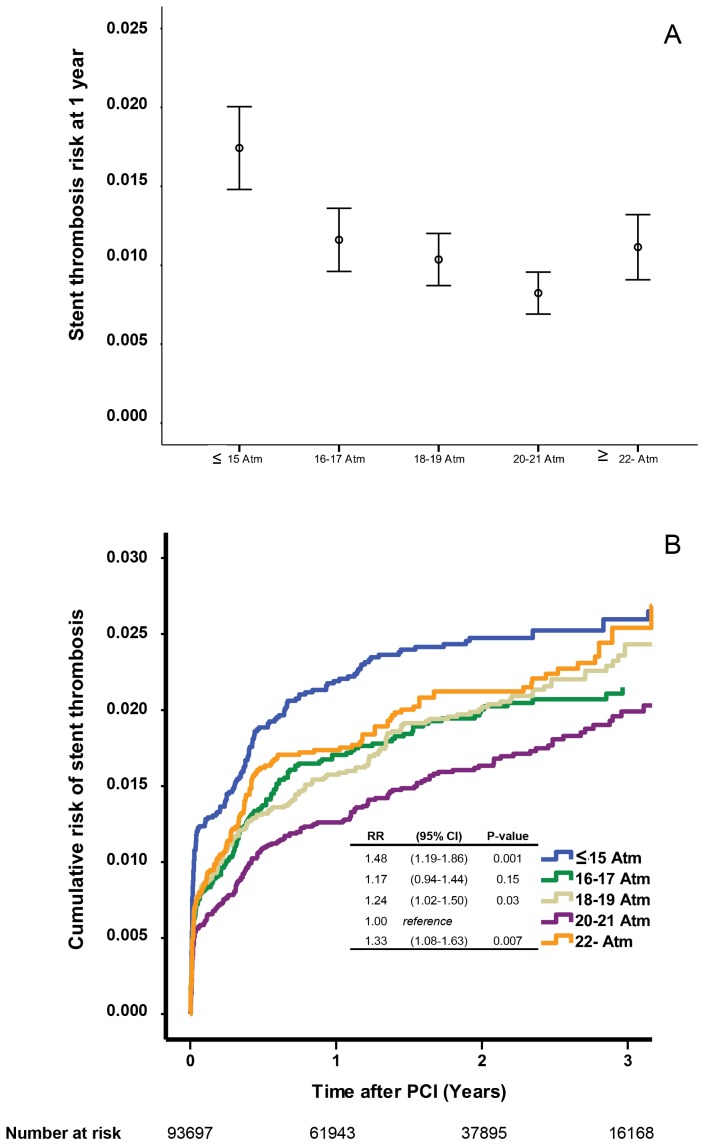
The risk of stent thrombosis at 1 year after PCI in relation to stent inflation pressure (panel A). Estimated cumulative event rates of stent thrombosis in relation to stent inflation pressure. RR stands for risk ratio and CI for confidence interval (panel B).

During the study period 999 stent thromboses were reported. The one-year incidence and the cumulative incidence of stent thrombosis in relation to stent inflation pressure are depicted in Figure 1A and 1B, respectively. With the 20–21 atm group as reference the risk of stent thrombosis was significantly higher in the ≤15 atm, 18–19 atm and ≥22 atm groups (Figure 1B).

In order to rule out possible bias related to a “per stent” analysis we repeated the analysis for patients stented for the first time and only receiving a single stent. In this group of 27 893 patients 284 stent thromboses were reported. Also with the 20–21 atm group as reference the risk ratios (RR) of stent thrombosis at one year were: ≤15 atm: RR 1.28 (95% confidence interval (CI) 0.85–1.95 P = 0.24); 16–17 atm: RR 1.06 (CI: 0.72–1.56, P = 0.79); 18–19 atm: RR 0.92 (CI: 0.63–1.35, P = 0.68); ≥22 atm: RR 1.36 (CI: 0.93–2.00, P = 0.12).

### Restenosis

**Figure 2:**
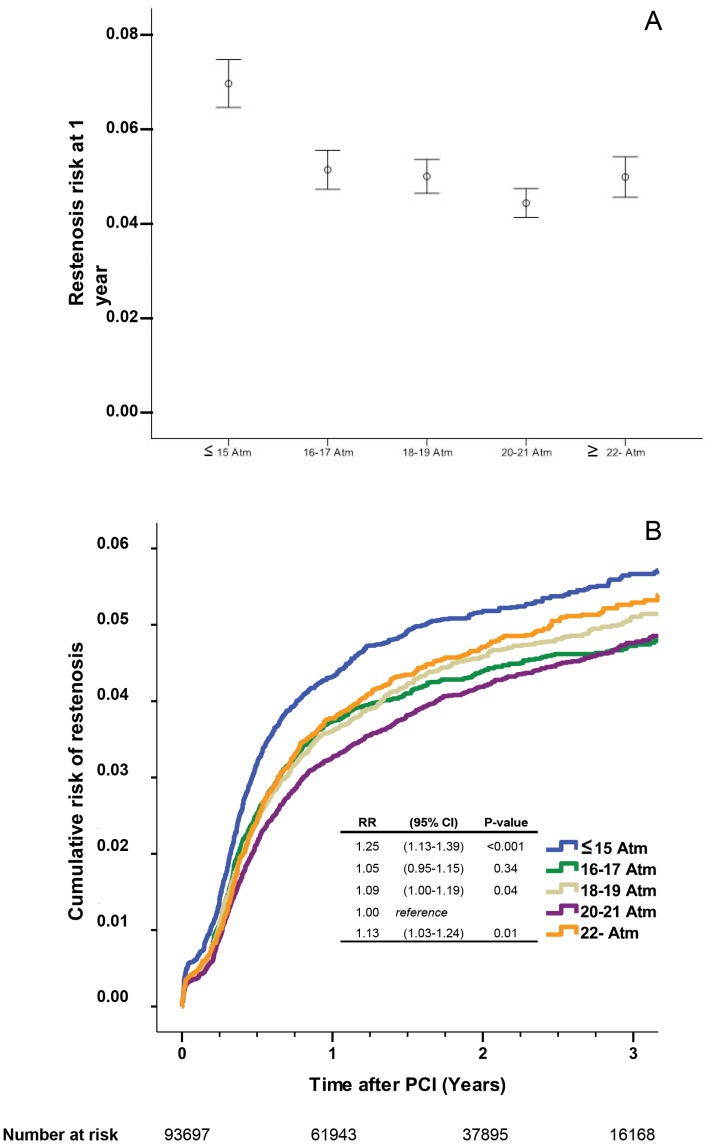
The risk of restenosis at 1 year after PCI in relation to stent inflation pressure (panel A). Estimated cumulative event rates of restenosis in relation to stent inflation pressure (panel B).

Restenosis was reported in 4 773 stents. The one-year incidence and the cumulative incidence of restenosis in relation to stent inflation pressure are depicted in Figure 2A and 2B, respectively. The risk of restenosis was significantly higher in the ≤15 atm, 18–19 atm and ≥22 atm groups (Figure 2B).

We also analysed the 27 893 patients stented for the first time and only receiving a single stent. In this group of patients 1178 restenoses were reported. With the 20–21 atm group as reference the RRs of restenosis at one year were: ≤15 atm: RR 1.31 (CI: (1.07–1.62) P = 0.010); 16–17 atm: RR 1.08 (CI: 0.90–1.31, P = 0.41); 18–19 atm: RR 1.12 (CI: 0.94–1.33, P = 0.22); ≥22 atm: RR 1.27 (CI: 1.05–1.53, P = 0.016).

### Mortality

**Figure 3:**
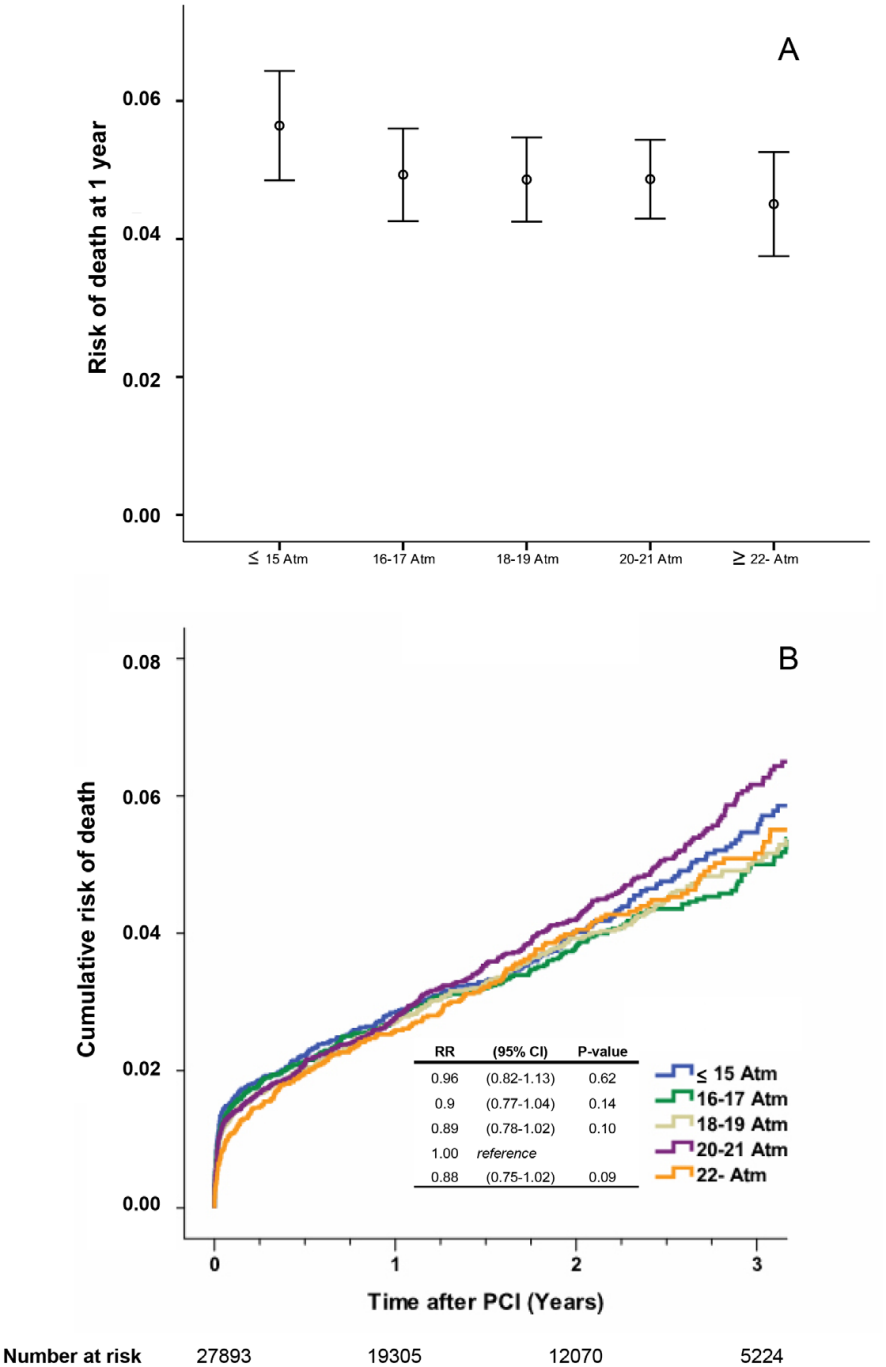
The risk of death at 1 year after PCI in relation to stent inflation pressure (panel A). Estimated cumulative event rates of death in relation to stent inflation pressure (panel B).

In the group of patients receiving a single stent only (27 893 patients) 1 902 deaths were reported. The one-year mortality and the cumulative incidence of death in relation to stent inflation pressure are depicted in Figure 3A and 3B, respectively. There were no statistically significant differences in death between groups.

### Low versus high balloon inflation pressure

Clinically and considering the imprecision of balloon inflation device manometers it could be reasoned that a division into “low” and “high” balloon inflation pressures would make the findings easier to interpret from an individual patient's point of view. We defined a low balloon inflation pressure as ≤18 atm (50 665 stents) and a high pressure as ≥19 atm (43 032 stents). The RR risk for stent thrombosis demonstrated a statistically non-significant trend towards increased risk with a low balloon pressure (RR 1.14 (CI: 0.98–1.32) P = 0.084). For restenosis (RR 1.05 (CI: 0.98–1.12) P = 0.16) and mortality (RR: 0.94 (CI 0.85–1.05) P = 0.27) no differences were found.

### Post-dilatation

**Figure 4:**
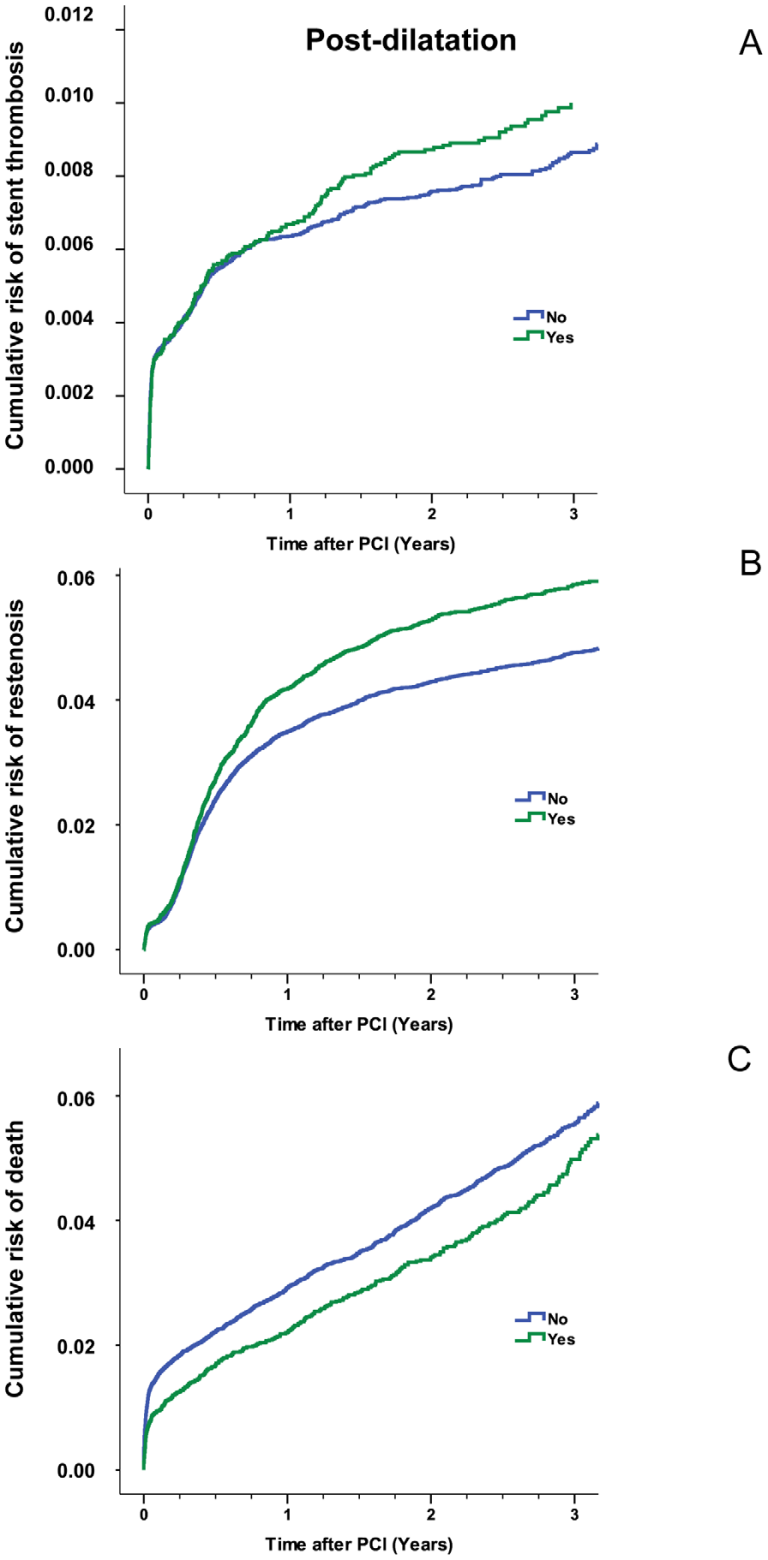
Estimated cumulative event rates of stent thrombosis in relation to post-dilatation (panel A). The RR of stent thrombosis of 1.18 (CI 0.95–1.32) did not differ statistically between procedures with or without post-dilatation (P = 0.19). Estimated cumulative event rates of restenosis in relation to post-dilatation (panel B). Restenosis occurred more often following post-dilatation compared with procedures where this adjunct was not used (RR 1.22 (CI 1.14–1.32) P<0.001). Estimated cumulative event rates of death in relation to post-dilatation (Panel C). The risk of death was lower following post-dilatation (RR 0.81 (CI 0.71–0.93) P = 0.003). The numbers at risk are for stent thrombosis and restenosis are identical to Figure 1 and 2 while the numbers at risk for death are identical to Figure 3.

Overall, post-dilation was not associated with a statistically significant lower risk of stent thrombosis (Figure 4A). Restenosis was more often seen following post-dilatation and this reached statistical significance (Figure 4B). For both variables the Kaplan-Meier curves separated after approximately one year. Conversely, mortality was higher in patients where post-dilatation was not performed and the curves separated shortly after PCI (Figure 4C).

Because the most optimal stent inflation pressure with respect to stent thrombosis and restenosis appeared to be 20–21 atm we did a separate analysis of the effect of post-dilatation within this pressure interval which is routinely used both as the highest inflation pressure during stenting with and without post-dilatation. In this analysis the use of post-dilatation was associated with a RR of stent thrombosis at one year of 1.56 (CI: (1.10–2.23) P = 0.013), the RR of restenosis was 1.15 (CI: (0.98–1.34) P = 0.079) and the RR of death was 0.89 (CI: (0.71–1.12) P = 0.330).

### Possible interactions

There was no statistically significant interaction between indication for PCI (stable angina pectoris, non-ST segment myocardial infarction or ST-segment myocardial infarction) and endpoints (stent thrombosis, restenosis or death) related to stent inflation pressure. Likewise, there was no statistically significant interaction between post-dilatation and type of stent (bare metal or drug-eluting) and endpoints related to stent inflation pressure. For the 3 different end-points stent thrombosis, restenosis and death the interaction P-values were: for pressure*indication 0.241, 0.163 and 0.363, for pressure*post-dilatation 0.818, 0.465 and 0.784, and for pressure*type of stent 0.609, 0.709 and 0.871, respectively.

## Discussion

### Summary of findings

In this retrospective analysis of stent inflation pressure and clinical outcome of more than 90 000 stents a biological pattern emerged - the risks of stent thrombosis and of restenosis appeared to be higher with low and very high pressures. Contrary to our expectation post-dilatation was associated with a higher risk of restenosis and a lower risk of death immediately following PCI.

### Atmospheres – what is the ideal number?

In modern PCI malapposition and underexpansion of stents are considered major risk factors for stent thrombosis [12] and this view has been supported by observational studies using different imaging techniques [13],[14] and confirmed in a case-control study using IVUS [15]. Such findings are probably the main reason for the use of high inflation pressures during stent implantation despite the lack of documentation from prospective trials. However, there is more to a coronary artery than the interaction between stent and intimal layer. We previously demonstrated that atherosclerotic coronary artery distensibility by balloon inflation is a linear function of pressure at low inflation pressures only and primarily in arteries with concentric lesions [16]. At higher pressures, of relevance for PCI, distensibility was unpredictable. It complicates matters even more for the operator that manufacturer stent balloon compliance charts grossly overestimate the final stent dimensions, as these measurements are typically made in water without the vessel constraint that limits balloon expansion [17]. Very high stent inflation pressures may cause stent edge dissection, coronary rupture, media and intima rupture leading to an increased inflammatory response and higher restenosis rate [18] [19] – factors that may help to explain our findings in the ≥22 atm pressure group. Our study identified a possible optimal stent inflation pressure of 20–21 atm during PCI, which was associated with a lower risk of stent thrombosis and restenosis – a finding that fits well with the studies cited above.

### Post-dilatation

Stent balloons are usually semi-compliant but an optimal stent expansion as documented by intravascular ultrasound cannot be ensured by inflation to very high pressures. Typically, high inflation pressures of semi-compliant stent balloons result in earlier opening, larger diameter and thus increased wall stress in the extreme proximal and distal ends during stent expansion – so-called “dogboning” [20]. Based on smaller studies but without offset in randomized trials post-dilatation with an NC balloon to ensure optimal stent expansion has been a standing recommendation within the PCI community.

However, post-dilatation is not without risks. The procedure involves advancement of yet another catheter and accurate placement of the NC balloon within the borders of the stent is not always achieved and this may result in edge dissection, geographic miss [21], or even coronary perforation [22]. These complications typically lead to additional stenting or target lesion revascularization at a later stage. Another possible complication with post-dilatation is longitudinal stent deformation – a problem which seems more likely with conformable newer generation stents with thin struts [23].

Our findings of a higher restenosis risk following post-dilatation was remarkable and the gradual and continuing separation of the Kaplan-Meier curves (Figure 4B) points towards a biological explanation. One explanation could be that post-dilatation in itself is injurious. Another possible explanation could be that operators tend to use this adjunct in PCIs of lesions confined with a known increased risk of restenosis – e.g. restenotic, long or calcific lesions, small vessels, bifurcations, chronic total occlusions or lesions in patients with diabetes. However, all of these factors were forced into our propensity score method and this considerably reduces the likelihood that this explains our findings.

The lower mortality seen with post-dilatation was almost immediate following PCI and there was no additional separation of curves over time (Figure 4C). It is probable that this reflects factors not directly related to post-dilatation and was due to selection bias not accounted for in our Cox proportional hazard regression model. This notion is supported by the finding that early stent thrombosis did not differ between groups (Figure 4A).

### Limitations

Despite appropriate statistical adjustments it is possible that unknown confounders may have affected the results of this registry study. The definition of stent thrombosis in our material is not identical to the definition set by the Academic Research Consortium (ARC). The SCAAR definition corresponds to definite stent thrombosis but excludes non-occlusive stent thrombosis. The SCAAR does not hold information on the duration of stent inflation, a parameter closely related to proper stent expansion [24]. The ratio between vessel diameter and balloon diameter was not available and neither was the number of post-dilatations. Another important parameter for this study, not registered in SCAAR, is degree of calcification as calcification per se could be a reason to post-dilate a stent. In SCAAR, when an operator indicates post-dilatation in the database there is no option to specify whether an NC or semi-compliant balloon is used. Our impression is that only a small subset of post-dilatations is done with semi-compliant balloons but the exact numbers are unknown and this must be taken into account when interpreting our results.

When analyzing data from very large databases, like SCAAR, there is a risk of finding statistically significant differences which do not translate into biologically meaningful information. We have tried to avoid this by viewing the data in different ways both depicting cumulative incidences and risks at 1 year and performing separate analyses in patients stented for the first time and only receiving a single stent. In our view the message lingering is that there could be increased risks of restenosis and stent thrombosis at pressure extremes and adjunct post-dilatation could be associated with an increased risk of restenosis. It is important to consider that PCI operators decision of balloon inflation pressure and whether or not to use post-dilatation cannot be considered subjective choices but are largely driven by achieving the best possible angiographic results. This interplay between plaque composition and operator decision cannot be deducted from our data. However, all different lesion subsets were included and analysed in the adjusted analyses but our findings must be interpreted with a grain of salt because of known and unknown factors not adjusted for in our statistical model. Further studies are therefore needed to crack the possible biological and clinical impact.

### Conclusions and clinical implications

This retrospective study of 93 697 stent implantations representing all eligible procedures in Sweden during more than 3.5 years identified a possible biological pattern - the risks of stent thrombosis and of restenosis appeared to be higher with low and very high pressures. Despite statistical adjustment we found a higher restenosis risk following post-dilatation. Post-dilatation was also associated with a lower mortality directly following PCI but the lack of further separation of survival curves over time hints to selection bias. Unmeasured residual confounding factors could partly explain our findings which warrant testing in a prospective, randomized trial.
